# Autophagy in the lung: guardian of homeostasis or driver of disease

**DOI:** 10.1080/27694127.2025.2568537

**Published:** 2025-10-09

**Authors:** Hyungsin Kim, Wenping Wang, Ioana Dobrescu, Joel Lee, Joshua Martorelli, Samuel Wang, Jessie Yanxiang Guo

**Affiliations:** a Rutgers Cancer Institute, New Brunswick, NJ, USA; b Department of Medicine, Robert Wood Johnson Medical School, Rutgers University, New Brunswick, NJ, USA; c Department of Chemical Biology, Rutgers Ernest Mario School of Pharmacy, Piscataway, NJ, USA

**Keywords:** Autophagy, metabolism, lung homeostasis, lung disease, lung disease treatment

## Abstract

Autophagy is a lysosome-directed recycling program that preserves lung homeostasis yet, when dysregulated, can cause disease. This review organizes current evidence by lung compartment and disease phase, proposing that autophagy polarity is determined by cell identity, micro-niche, and timing along the injury-repair continuum. In chronic obstructive pulmonary disease, epithelial autophagy is initially cytoprotective, but chronic smoke exposure reveals a lysosome bottleneck and stalled flux, while alveolar macrophages show impaired xenophagy and poor acidification. In idiopathic pulmonary fibrosis, autophagy is suppressed in type II epithelial cells and fibroblasts downstream of transforming growth factor beta (TGF-β) and mTORC1, which promotes epithelial stress programs and collagen translation. In acute lung injury and respiratory distress syndrome, timely autophagy activation limits cGAS-STING and NLRP3 signaling, preserves barrier integrity, and supports recovery. In asthma, autophagy supports mucin biogenesis in epithelial cells but is reduced in antigen-presenting cells, while eosinophil and mast cell effector functions rely on autophagy. In infection, xenophagy clears microbes but is actively subverted by bacteria and respiratory viruses. In non-small cell lung cancer (NSCLC), tumor-intrinsic autophagy maintains energy metabolism, redox balance, and enables immune evasion, whereas host autophagy can alternately support antitumor immunity or supply nutrients. We summarize small-molecule modulators, delivery strategies, and flux-aware tools that enable precise, cell- and phase-resolved modulation of autophagy to guide patient selection and improve therapy in respiratory disease.

## Introduction

The lungs are continuously exposed to inhaled particulates, microbial pathogens, allergens, and environmental toxins^[1–5]^. To maintain cellular and tissue homeostasis in this hostile environment, pulmonary cells rely on robust quality control mechanisms^[6–8]^. Among these, Macroautophagy (hereafter referred to as autophagy) plays a central role by degrading damaged organelles, misfolded proteins and intracellular pathogens via lysosome-mediated recycling. This process is essential for maintaining redox balance, genomic stability, proteostasis, and energy homeostasis^[9–15]^.

Autophagy is initiated by the formation of double-membrane autophagosomes that sequester cytosolic components and deliver them to lysosomes for degradation and intracellular recycling^[16,17]^. The pathway is regulated by nutrient- and stress-sensing kinases, particularly mechanistic target of rapamycin complex 1 (mTORC1) and AMP-activated Protein Kinase (AMPK), and interfaces with specialized forms such as mitophagy and chaperone-mediated autophagy^[18–21]^. Although basal autophagy is active in most lung cell types under physiological conditions, including alveolar epithelial cells, macrophages, and endothelial cells, its activity is dynamically regulated by metabolic cues, immune stimuli, and environmental stressors^[22–25]^.

Accumulating evidence implicates the importance of autophagy in both lung homeostasis and disease^[26,27]^. Depending on the context, autophagy may confer cytoprotection or drive pathology^[28]^. This review summarizes the cell-type-specific roles of autophagy in maintaining pulmonary function and evaluates how altered autophagy states contribute to major lung diseases, including chronic obstructive pulmonary disease, pulmonary fibrosis, acute lung injury, asthma, lung infections, cancer, cystic fibrosis, and pulmonary hypertension. We further highlight therapeutic strategies and emerging technologies aimed at modulating autophagy in the lung, and outline future directions for precision targeting of autophagy in respiratory disease.

### Autophagy pathway and its pharmacologic control in the lung

#### Autophagy regulation and processing

**Figure 1.**
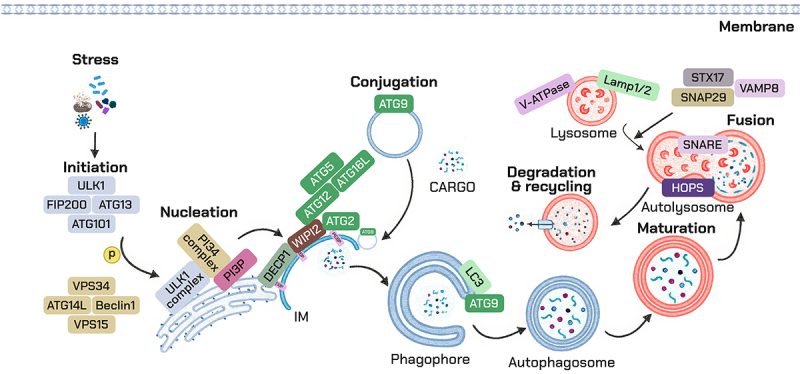
The autophagy pathway and selective variants relevant to the lung. Stepwise pathway from initiation to degradation. ULK1 complex recruits the VPS34 complex I to generate PI3P at the phagophore; WIPI2 and ATG2 scaffold membrane expansion; ATG12–ATG5–ATG16L1 and ATG8/LC3 conjugation drive elongation and closure; STX17–SNAP29–VAMP8 and HOPS mediate autophagosome–lysosome fusion; v-ATPase acidification activates cathepsins and LIPA to complete degradation.

Autophagy is a highly conserved cellular process, with its activation orchestrated by a network of >30 core autophagy-related genes (ATG) first discovered in yeast and largely conserved in mammals^[29–33]^. Autophagy is tightly regulated by nutrient and energy availability via the opposing activities of mTORC1 and AMPK. Under nutrient-rich conditions, mTORC1 functions as the master-negative regulator by inhibiting the Unc-51-like kinase 1 (ULK1) complex, thereby suppressing autophagy initiation. In contrast, when cellular energy levels are low, AMPK is activated in response to increased AMP/ATP ratios; it directly phosphorylates and activates ULK1 while simultaneously inhibiting mTORC1, promoting autophagy induction^[34,35]^. Autophagosome initiation is triggered by the ULK1 complex (ULK1, ATG13, RB1CC1/FIP200, ATG101), which activates the class III phosphatidylinositol 3-kinase (PI3K) complex I (PIK3C3/VPS34, BECN1, ATG14, AMBRA1) to generate phosphatidylinositol 3-phosphate and recruit membrane effectors such as WD‑repeat phosphoinositide‑interacting protein 2 (WIPI2) and double FYVE domain-containing protein 1 (DFCP1)^[36,37]^. Phagophore expansion requires the ATG9A membrane source and two ubiquitin-like conjugation systems: ATG7 and ATG3 load microtubule-associated protein 1A/1B-light chain 3 (LC3)/gammaaminobutyric acid receptor-associated protein (GABARAP) family members onto phosphatidylethanolamine, while the ATG12–ATG5–ATG16L1 complex acts as an E3-like ligase to localize LC3 lipidation to the growing rim^[38,39]^. Cargo selection is mediated by receptors including sequestosome-1 (SQSTM1)/p62, BRCA1 gene 1 (NBR1), optineurin (OPTN), nuclear dot protein 52 (NDP52) and Tax1-binding protein 1 (TAX1BP1), which bind ubiquitinated substrates and LC3 family proteins^[40]^. Maturation and trafficking depend on Rab7 GTPase (RAB7) and the homotypic fusion and vacuole protein sorting (HOPS) tethering complex, with autophagosome-lysosome fusion executed by the syntaxin 17 (STX17)– synaptosomal‑associated protein 29 (SNAP29)–vesicle‑associated membrane protein 8 (VAMP8) soluble Nethylmaleimide–sensitivefactor attachment protein receptors (SNAREs)^[41]^. Degradation requires an acidic, hydrolase-rich lysosome maintained by the vacuolar H^+^-ATPase (VATPase) and lysosome-associated membrane protein 1/2 (LAMP1/2), with cathepsins B, D and L among the principal proteases. Transcriptional control of lysosome-and autophagy genes is coordinated by Transcription factor EB (TFEB) and transcription factor E3 (TFE3)^[42]^ (Figure 1). As detailed in Figure 23, lysosomal signaling integrates nutrient and redox inputs: mTORC1 and AMPK act in opposition to control ULK1 activity and TFEB/TFE3 nuclear translocation, while ROS fine‑tunes autophagy flux via AMPK activation, oxidation of ATG4, JNK –BECN1 engagement, and stabilization of PINK1.

**Figure 2.**
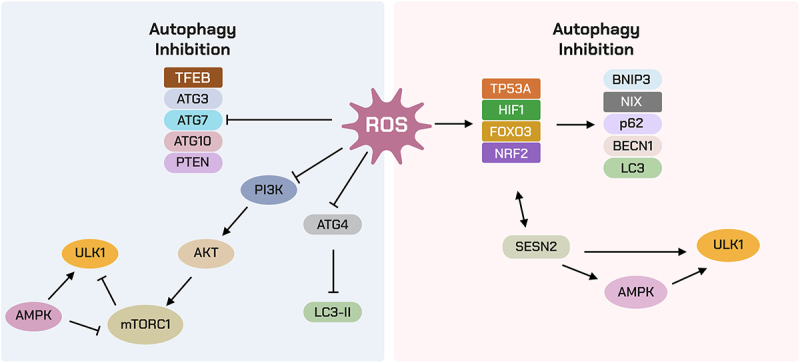
Regulatory surface at the lysosome. mTORC1 vs AMPK control ULK1 and TFEB/TFE3; ROS inputs tune AMPK, ATG4 oxidation, JNK–BECN1, and PINK1 stabilization.

### Selective autophagy modalities

**Figure 3.**
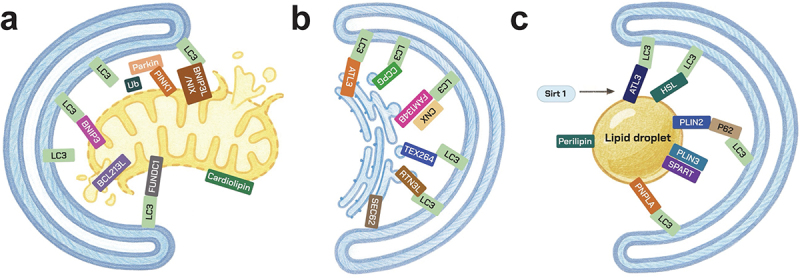
Selective autophagy nodes engaged in lung disease. a. Mitophagy via PINK1–PRKN and BNIP3/NIX. b. ER-phagy via FAM134B, RTN3L, CCPG1, SEC62, TEX264. c. lipophagy via p62/NBR1 and RAB7–dependent droplet–lysosome contacts.

Selective autophagy represents a spectrum of specialized pathways that enable cells to precisely remove damaged or superfluous components while maintaining homeostasis (Figure 3). Among the best characterized is mitophagy, which eliminates dysfunctional mitochondria through regulators such as PTEN-induced kinase 1 (PINK1), Parkin, BCL2/adenovirus E1B 19 kDa interacting protein 3 (BNIP3), and BNIP3-like (BNIP3L, also called NIX), thereby preserving mitochondrial quality control and limiting oxidative stress^[43,44]^. Other organelles are targeted through pathways such as pexophagy for peroxisomes and ER-phagy for the endoplasmic reticulum (ER), mediated by receptors including family with sequence similarity  134 member B (FAM134B) and reticulon -3 (RTN3)^[45,46]^. Protein aggregates are cleared through aggrephagy, which relies on receptors such as p62 and NBR1^[47]^, while lipophagy mobilizes lipid droplets to regulate energy metabolism^[48]^. Autophagy also contributes to innate immunity through xenophagy, which eliminates intracellular pathogens including *Salmonella* and *Listeria* ^[49]^. Additional specialized forms include ferritinophagy, which liberates iron from ferritin via the nuclear receptor coactivator 4 (NCOA4)^[50]^; ribophagy, which selectively degrades ribosomes^[51]^; and nucleophagy, which removes nuclear material^[52]^. Collectively, these processes underscore the versatility of autophagy as both a quality control and adaptive mechanism, with broad implications for metabolism, infection, and disease.

### Small-molecule agents applied in pulmonary studies to control autophagy

A variety of small-molecule agents are used in pulmonary research to modulate distinct stages of the autophagy pathway. Rapamycin and metformin promote autophagy initiation by relieving mTORC1ULK1 inhibition or by activating AMPK; spermidine induces autophagy through epigenetic and translational control; hydroxychloroquine (HCQ) and chloroquine (CQ) raise lysosomal pH and block autophagosome–lysosome fusion; bafilomycin A1 inhibits the VATPase and fusion; and ULK1 and VPS34 inhibitors block autophagosome formation. In the disease sections that follow, we highlight how these agents align mechanistically with activated, inhibited or stalled autophagy states in each pulmonary context^[53,54]^ (Table 1).

**Table 1.** Small molecule autophagy modulators discussed in this review and their pulmonary relevance.

Agent or class	Primary autophagy node & mechanism	Direction of autophagy	Pulmonary disease context & translational note	References
Rapamycin (sirolimus)	mTORC1 inhibition releases ULK1 & initiates autophagy	Inducer	IPF: reduces fibroblast activation & epithelial stress in models; ALI or ARDS: lowers IL1β & IL18 & improves permeability; CF: improves macrophage bacterial clearance in ex vivo cells & models; inhaled delivery under study	89, 110, 195, 201, 176, 215–217
Metformin	AMPK activation with secondary mTORC1 relief	Inducer	IPF: reinstates flux & attenuates matrix programs in preclinical studies; broader lung deployment remains investigational	89, 99, 195
Spermidine	Epigenetic promotion of autophagy gene expression & TFEB activity	Inducer	Anti-inflammatory & anti-fibrotic signals in preclinical systems; translational studies in lung disease are early	196, 197
HCQ	Lysosome alkalinization & reduced autophagosome to lysosome fusion	Inhibitor	NSCLC: used as an autophagy blocker in combination strategies; may sensitize to targeted therapy but can impair response to anti-PD-1 in some settings, so trial design should be cautious	198, 200, 202, 209
Bafilomycin A1	VATPase inhibition prevents acidification & fusion	Inhibitor	Research tool to confirm flux blockade in lung models; not for clinical use	199
ULK1 inhibitors	Block autophagosome initiation at ULK1 or ULK2	Inhibitor	KL NSCLC: restore antigen presentation & improve response to anti-PD-1 & to MEK inhibition in vivo; prospective clinical selection with ULK1 high modules is being explored	160, 162, 163
VPS34 inhibitors	Block PI3KC3 Vps34 complex & phagophore nucleation	Inhibitor	Preclinical autophagy blockade in cancer & inflammatory models; candidate for NSCLC combinations where flux is tumor supportive	198
Cysteamine	Inhibits TGM2 to liberate Beclin1 & restore BECN1–VPS34 function	Inducer	CF epithelium: rescues autophagy execution & improves CFTR trafficking; combinable with CFTR modulators & EGCG	177, 178
Epigallocatechin gallate (EGCG)	Antioxidant & lysosome supportive cofactor that stabilizes corrected CFTR	Inducer	CF epithelium: augments cysteamine effects & normalizes proteostasis; candidate for airway targeted delivery	177, 178
Vitamin D3	Promotes antimicrobial autophagy programs in innate cells	Inducer	Infectious lung disease & tuberculosis: enhances xenophagy & bacterial clearance in experimental systems	211

## Autophagy in normal lung homeostasis

Autophagy is essential for lung homeostasis under basal conditions and is active across multiple pulmonary cell types, including alveolar epithelial cells, alveolar macrophages, club cells, and vascular endothelium. In each context, autophagy coordinates metabolic adaptation, organelle turnover, and stress resilience to preserve pulmonary function^[27,55,56]^.

In alveolar macrophages, autophagy limits oxidative stress and excessive inflammation by clearing phagocytosed cargo, damaged mitochondria, and inflammasome components. Loss of autophagy in macrophages impairs pathogen clearance, exacerbates reactive oxygen species (ROS) accumulation, and enhances IL-1β and tumor necrosis factoralpha (TNF-α) production, leading to tissue damage and remodeling^[56–61]^.

Alveolar type II (AT2) epithelial cells depend on autophagy to maintain surfactant homeostasis^[62,63]^. Autophagy regulates turnover of lamellar bodies and prevents accumulation of dysfunctional surfactant complexes. Disruption of autophagy in AT2 cells induces ER stress and impairs alveolar compliance, predisposing the lung to collapse and injury^[64–66]^.

Multiciliated epithelial cells rely on mitophagy to sustain mitochondrial fitness and ciliary function^[67,68]^. Loss of mitophagy compromises ATP production and disrupts ciliary beating, impairing mucociliary clearance and host defense^[69–71]^.

During postnatal development, autophagy is upregulated to support alveolarization, epithelial differentiation, and tissue remodeling^[72,73]^. In contrast, aging is associated with a decline in autophagic flux, leading to the accumulation of damaged organelles, impaired regeneration, and increased susceptibility to infection and fibrosis^[74–77]^.

Collectively, these findings establish autophagy as a constitutive quality control mechanism required for epithelial integrity, immune homeostasis, and organ-level resilience in the lung. Disruption of autophagy in any of these compartments compromises lung defense and predisposes to disease.

## Autophagy in lung diseases

Autophagy’s apparent “two faces” are not paradoxical, but state‑dependent outcomes of a pathway whose inputs, cargo, and execution machinery are wired differently across lung cell types, microenvironments, and disease phases^[27]^. Whether autophagy is protective or pathological in the lung is largely determined by three axes: phase of stress (short‑term activation that clears the right cargo is protective, chronic injury often reveals an execution bottleneck at the lysosome and becomes harmful)^[11]^; cell identity and tasking (for example, AT2 cells require autophagy for surfactant quality control, pulmonary artery smooth muscle cells (PASMCs) use it to sustain hypoxic proliferation, endothelium needs basal flux for endothelial nitric oxide synthase (eNOS) coupling, macrophages rely on xenophagy for host defense)^[66]^; and micro‑niche cues (cytokines, hypoxia, metabolic substrate availability) that alter cargo selection and TFEB/TFE3‑driven lysosome capacity^[78]^. Here we specify activated, inhibited, or stalled states by cell type and disease stage and connect them to agent classes that either restore autophagy flux or strategically constrain it.

For clarity, each disease subsection is organized by the major lung compartments. Where evidence allows, we state whether autophagy is activated, inhibited, or stalled at completion, list proximal mechanisms and functional readouts, and link to aligned small‑molecule logic. See Table 2 for a compact cell‑type‑by‑phase summary.

**Table 2.** Autophagy polarity by cell type and disease phase in the lung.

Disease	Cell type	Disease phase	Autophagy state	Proximal mechanism (concise)	Functional consequence	Representative markers and readouts	References
COPD	Airway epithelium	Early smoke exposure	Activated	Sestrin, AMPK, and ULK1 engagement with relief of mTORC1 restraint; efficient mitophagy and lysosome function	Cytoprotection, lower mitochondrial reactive oxygen, restraint of cGAS and STING and NLRP3	Transient rise in LC3 II with flux, lower p62 or SQSTM1, reduced IL-1β and IL18	52–72
	Airway epithelium	Chronic smoke exposure	Stalled at completion	Lysosome acidification failure with reduced ATPase and cathepsin activity; TFEB program insufficient to scale	Ciliary loss, mucus hypersecretion, apoptosis, airway remodeling	Accumulation of p62 or SQSTM1 and ubiquitinated cargo, LC3 II accumulation without turnover, reduced LAMP1 colocalization	
	Alveolar macrophage	Chronic smoke exposure	Stalled xenophagy	Poor phagolysosome acidification; persistence of damaged mitochondria and cytosolic mitochondrial DNA, activation of cGAS and STING and NLRP3	Diminished bacterial killing, chronic colonization, sustained inflammation	Low LC3 recruitment to phagosomes, low LysoTracker signal, elevated IL-1βand IL-18	
	Epithelium and macrophage	Chronic stress	Maladaptive mitophagy and necroptosis bias	Persistent PINK1 and Parkin signaling depletes healthy mitochondria and lowers threshold for RIPK1, RIPK3, and MLKL	Necroptosis, DAMP release, emphysema features	PINK1 and PRKN elevation, phospho MLKL, HMGB1 in lavage	
IPF	AT2 epithelium	Progressive fibrosis	Inhibited	Beclin1 and VPS34 impairment with p62 accumulation; unresolved endoplasmic reticulum stress; p62, NF-κB, and Snail axis	Mitochondrial dysfunction, epithelial to mesenchymal transition, profibrotic secretome	Low LC3 lipidation, high p62, CHOP or BiP elevation, transitional AT2 states	75–99
	Fibroblast	Progressive fibrosis	Inhibited	Transforming growth factor beta sustains mTORC1 and inhibits ULK1 initiation while promoting collagen translation	Myofibroblast persistence, matrix deposition, apoptosis resistance	Phospho S6, phospho ULK1 S757, COL1A1 translation, reduced LC3 flux	
	AT2 epithelium in aging	Aging bias	Inhibited	Downregulation of USP13 destabilizes Beclin1	Senescence and exaggerated fibrosis after injury	Low USP13, high p16 and p21, low LC3 flux	
ALI or ARDS	Macrophage	Early sepsis or endotoxin	Activated when protective	Mitophagy removes damaged mitochondria and limits mitochondrial DNA release, restraining cGAS and STING and NLRP3	Lower IL-1β and IL-18, reduced permeability and hypoxemia	Increased LC3 puncta with flux, reduced caspase-1 activity, lower IL-1β	80, 100–113
	Alveolar epithelium	Injury and recovery	Activated when protective	Autophagy sustains tight junctions and reduces endoplasmic reticulum stress; during recovery restrains PI3K, AKT, and mTOR	Barrier preservation; AT2 proliferation and differentiation	Preserved ZO-1, lower CHOP, increased LC3 flux during repair	
Asthma	Airway epithelium	Type 2 high endotype	Activated but poorly completed	Interleukin 4 and interleukin 13 drive mucin biogenesis and secretory organelle turnover without efficient completion	Mucus accumulation and barrier decline	LC3 puncta with p62 buildup, high MUC5AC, reduced tight junction proteins	114–126
	Dendritic cell and macrophage	Type 2 high endotype	Inhibited	Interleukin 4 and interleukin 13 reduce autophagy in antigen presenting cells	Prolonged antigen presentation and Thelper 2 priming	Lower LC3 flux, sustained MHC II display, higher costimulatory signaling	
	Eosinophil and mast cell	Exacerbation	Autophagy dependent effector programs	Core ATG proteins required for extracellular trap formation, degranulation, and granule maturation	Airway inflammation and hyperreactivity	Genetic loss of ATG5 or ATG7 reduces EETs and degranulation; altered tryptase and cytokines	
	Macrophage	Alarmin dominated vs microbial	Context dependent	Interleukin 33 and interleukin 25 promote mitophagy and M2 repair; microbial signals couple autophagy to NLRP3 and T-helper 1 and T- helper 17	Repair in alarmin context; steroid resistance with microbial co signals	BNIP3 or BNIP3L expression, NLRP3 and IL-1β elevation in microbial context	
Pulmonary infections	Macrophage in Mtb	Active infection	Activated or subverted	Interferon gamma induces p62, NDP52, and optineurin and promotes fusion; virulent strains activate AKT and mTOR and block HOPS and SNARE assembly	Pathogen clearance when engaged; intracellular survival when subverted	LC3 and LAMP1 colocalization when effective; fusion block with stalled LC3	127–140, 173–176
	Alveolar macrophage in Pseudomonas or Burkholderia	Persistent infection	Stalled xenophagy	Acidification failure and poor LC3 recruitment to pathogen vacuoles	Intracellular persistence and tissue injury	Low LysoTracker, low LC3 recruitment, high cGAS and STING and NLRP3	
	Airway or alveolar epithelium in viral disease	Acute viral pneumonia	Subverted	Viral proteins reduce Beclin 1 or block fusion and acidification, for example SARS-CoV-2 ORF3a	Enhanced replication and epithelial injury	Reduced LC3 and LAMP1 colocalization, Beclin 1 decline, ORF3a expression	
Lung cancer	Tumor cell, premalignant	Initiation	Activated and tumor suppressive	Autophagy removes damaged mitochondria and prevents p62 accumulation	Lower oxidative DNA damage and delayed initiation	Low p62, lower 8-oxo-dG, intact LC3 flux	141–165
	Tumor cell, established KRAS	Progression	Activated and required	Autophagy maintains tricarboxylic acid flux, nucleotide pools, and redox balance; reliance amplified with LKB1 loss	Tumor maintenance, therapy resistance, lower MHC-I presentation	Atg7 dependence in models, high ULK1 and TFEB modules, reduced MHC-I	
Cystic fibrosis	Airway epithelium	Chronic disease	Stalled at execution	Transglutaminase 2 sequesters Beclin 1 in aggresomes; Beclin 1 and VPS34 complex disabled	Proteotoxic stress and NF-κB activation; impaired CFTR trafficking	p62 and HDAC6 aggresomes, low LC3 flux, restored by cysteamine and EGCG	166–179
	Alveolar macrophage	Chronic infection	Inhibited xenophagy	Reduced phagolysosome acidification and LC3 recruitment; ATG12 epigenetic silencing	Persistent pathogens and amplified inflammasome activity	Low acidification, low LC3 on vacuoles, high IL-1βI; improved by interferon gamma or rapamycin	
Pulmonary hypertension	PASMC	Hypoxic remodeling	Activated and pro-proliferative	AMPK and mTOR adaptation sustains survival and migration	Medial thickening and vessel occlusion	Increased LC3 with flux, higher Ki-67, preserved mitochondria	182–193
	Endothelium	Chronic remodeling	Dual role	Basal autophagy preserves nitric oxide synthase coupling; chronic activation in specific segments promotes endothelial to mesenchymal transition	Vasodilation when preserved; plexiform lesions when overactivated in select territories	eNOS coupling and tetrahydrobiopterin indices, TFEB or FOXO1 programs, EndoMT markers	

### Chronic obstructive pulmonary disease (COPD)

COPD features progressive, largely irreversible airflow limitation with chronic bronchiolitis and emphysema most often caused by long-term cigarette smoke exposure, which establishes a persistent oxidative and proteotoxic milieu in the airway and alveolar units^[79,80]^.

Cigarette smoke delivers ROS, aldehydes, and metals that acutely activate autophagy in the airway and alveolar epithelium through sestrin-AMPK signaling with relief of mTORC1 inhibition^[79–82]^. In this early window, mitophagy selectively removes depolarized mitochondria, lowers mitochondrial reactive oxygen, and limits cyclic GMP-AMP synthase (cGAS)–stimulator of interferon genes (STING) and NLR family pyrin domain-containing 3 (NLRP3) activity^[83]^. Autophagy also clears carbonyl modified proteins and peroxidized lipids, maintains proteostasis, and supports epithelial viability^[61,82,84]^.

With persistent smoke exposure, lysosome capacity becomes limiting and autophagic flux is stalled, evidenced by accumulation of p62 and ubiquitinated cargo together with sustained endoplasmic reticulum (ER) stress and senescence^[85–89]^. In airway epithelium, impaired completion is linked to ciliary loss, mucus hypersecretion, and apoptosis, consistent with airway remodeling in human COPD^[90,91]^. These data support that the injurious phenotype reflects incomplete autophagy flux and lysosome dysfunction rather than simple overactivation.

Alveolar macrophages from patients show inhibited xenophagy and poor phagolysosome acidification, with persistence of damaged mitochondria, cytosolic mitochondrial DNA (mtDNA), and activation of cGAS–STING and NLRP3 that sustain interleukin-1 beta (IL-1β) and interleukin 18 (IL-18)^[92–94]^. Bacterial killing falls and colonization persists, providing human and *in vivo* support that impaired autophagy completion contributes to chronic inflammation and infection risk.

In mouse and cellular models, sustained activation of the PINK1–Parkin pathway can drive mitophagy beyond its normal role in quality control, leading to excessive clearance of healthy mitochondria and a lowered threshold for receptor-interacting serine/threonine protein kinase 1 (RIPK1)/receptor-interacting protein kinase 3 (RIPK3)/mixed lineage kinase domain-like pseudokinase (MLKL)-dependent necroptosis. This mitophagy-driven necroptotic mechanism has been demonstrated *in vivo* and linked to pathological features of emphysema^[95–97]^. This provides mechanistic evidence that mistuned mitophagy, not physiologic autophagy *per se*, can be pathological under chronic stress. Genetic variation in ATG5 and ATG16L1 further links autophagy capacity to COPD susceptibility^[84,98]^. The critical defect appears to lie in lysosomal function and completion of autophagic flux, rather than in indiscriminate autophagy induction. Accordingly, therapeutic strategies that restore TFEB-driven lysosomal biogenesis, enhance acidification, and promote selective mitophagy are most consistent with current evidence ^[79–82],[84–87], [89–101]^.

At present, no autophagy-targeted therapies are approved for COPD. Because the prevailing defect is impaired completion with lysosome dysfunction, lysosomotropic inhibitors such as HCQ or CQ would be expected to worsen cargo clearance and xenophagy. In contrast, strategies that stimulate autophagy initiation, such as AMPK activation by metformin or upstream mTORC1 inhibition by rapamycin, could in principle enhance flux. However, any such strategy should be tested with fluxcompetent biomarkers and with caution for increased infection risk^[102–107]^.

### Idiopathic pulmonary fibrosis (IPF)

IPF is a progressive fibrosing interstitial pneumonia with the usual interstitial pneumonia pattern and a median survival of three to five years, driven by repetitive epithelial injury and aberrant repair that activates fibroblasts and stiffens the lung^[108,109]^.

In fibrotic lungs, autophagy is inhibited at the Beclin1 and VPS34 complex and at the lysosome, with p62 accumulation and reduced LC3 lipidation in AT2 cells^[110–113]^. Failure to clear misfolded surfactant proteins sustains ER stress, mitochondrial dysfunction, and epithelial-mesenchymal transition through a p62- NF-κB-Snail axis^[114–117]^. Autophagy deficient epithelium secretes profibrotic cues that activate fibroblasts^[118–120]^.

In fibroblasts, TGF-β activates mTORC1, inhibits ULK1 mediated initiation, and increases cap-dependent translation, thereby coupling autophagy inhibition to collagen synthesis and apoptosis resistance^[98–102]^. These mechanisms have been reproduced in *ex vivo* human IPF tissues and models, and are reversed by ATP competitive mTOR inhibitors or AMPK activation, arguing for a causal role^[107,121–123]^.

Aging diminishes autophagy capacity in part through downregulation of ubiquitin specific peptidase 13 (USP13), which destabilizes Beclin1 in AT2 cells and amplifies senescence and fibrosis after injury in murine *in vivo* studies^[124–127]^. Together, cell identity and chronic TGF-β and mTOR wiring help explain the polarity of autophagy in fibrosis. Inhibited epithelial autophagy promotes transitional states and profibrotic secretomes, while reduced fibroblast autophagy sustains matrix-producing programs. Restoring flux through AMPK activation or mTORC1 blockade reestablishes protective autophagy in both compartments^[128–132]^.

In IPF, autophagy is inhibited in both epithelial cells and fibroblasts, accompanied by persistent mTORC1 signaling. Agents such as rapamycin and metformin align mechanistically with this defect by restoring autophagy initiation and reducing profibrotic collagen programs, consistent with experimental data from primary IPF fibroblasts and epithelial models. These compounds therefore represent rational, flux-restoring candidates for clinical study, provided that epithelial stress and immune competence are carefully monitored^[106,107,128–133]^.

### Acute lung injury (ALI)/acute respiratory distress syndrome (ARDS)

ALI and ARDS present with acute hypoxemic respiratory failure due to diffuse alveolar damage, capillary leak, and exuberant cytokine responses triggered by sepsis, pneumonia, trauma, or aspiration^[88,134,135]^.

In the context of sepsis, lipopolysaccharide activates Toll-like receptor 4 (TLR4)–myeloid differentiation primary response 88 (MyD88)–NF-κB signaling and promotes the assembly of NLRP3 inflammasomes, amplifying the inflammatory cascade. When autophagy is engaged, mitophagy selectively eliminates damaged mitochondria, thereby preventing the release of mtDNA and dampening downstream cGAS–STING and caspase-1 activation, which in turn reduces the production of IL-1β and IL-18^[88,134–142]^. Multiple *in vivo* studies show that pharmacologic autophagy induction reduces permeability and hypoxemia ^[136–138], [143]^.

In the alveolar epithelium, autophagy helps maintain barrier integrity by preserving tight junctions and alleviating ER stress. During the recovery phase, it supports AT2 cell proliferation and differentiation, in part through restraining PI3K-protein kinase B (AKT)-mTOR signaling^[116,144]^. Endothelium relies on basal autophagy to preserve nitric oxide synthase coupling and barrier integrity, while mesenchymal stem cell-derived vesicles activate autophagy programs and increase interleukin-10, thereby fostering endothelial protection and tissue repair^[145,146]^.

Lung injury is amplified when lysosomal acidification is impaired or when neutrophil extracellular traps accumulate beyond the capacity for resolution. Under these conditions, initiating autophagy without restoring lysosome competence proves ineffective^[137,147–149]^. Available evidence therefore supports timed, flux-competent activation rather than unselected induction^[88,134–146]^. Preclinical studies demonstrated that rapamycin attenuates inflammasome activation, vascular permeability, and hypoxemia when administered within a therapeutic window that preserves autophagic flux, supporting the concept that short-course, flux competent induction is protective in ALI/ARDS. In contrast, because lysosome competence is pivotal in severe injury, lysosomotropic autophagy blockers are unlikely to provide beneficial in this context ^[102–104],[136–138], [143]^.

### Asthma

Asthma comprises clinical and molecular endotypes ranging from type 2 high allergic disease to non-type 2 inflammation, unified by variable airflow obstruction, airway hyperresponsiveness, and mucus pathology^[150]^.

In airway epithelial cells, interleukin-4 (IL-4) and interleukin-13 (IL-13) activate autophagy, supporting mucin synthesis and secretory organelle turnover. When autophagic flux is inefficient, mucins accumulate and barrier proteins decline, facilitating allergen access and sustaining type 2 inflammation^[150–155]^. In antigen-presenting cells, IL-4 and IL-13 exert the opposite effect: autophagy is suppressed in dendritic cells and macrophages, prolonging antigen presentation and favoring T helper 2 priming^[151–154]^. Autophagy is also required for effector function in granulocytes; eosinophils require ATG proteins for extracellular trap formation, regulated degranulation, and survival, while mast cells require autophagy for granule maturation and cytokine release. These roles are supported by genetic loss of function studies^[156–159]^. Moreover, ORMDL sphingolipid biosynthesis regulator 3 (ORMDL3) restrains mast cell activation through an autophagy dependent ER stress program^[156]^. Epithelial alarmins such as interleukin-33 and interleukin-25 activate mitophagy in macrophages, lowering mitochondrial ROS and promoting M2 repair. Microbial signals instead couple macrophage autophagy to NLRP3 and T helper 1/17 responses, which are associated with steroid resistance^[151,160–162]^. Collectively, these findings illustrate that cytokine context and cell identity dictate whether autophagy serves to dampen or amplify airway inflammation.

Currently, there is no established use of autophagy modulators in asthma care. Given that epithelial autophagy can support mucin production, while autophagy in antigen-presenting cells is suppressed in type 2 inflammation, interventions such as mTORC1 inhibition or AMPK activation would likely require phenotype-guided deployment; lysosomotropic agents that impair antigen processing and host defense are unlikely to provide therapeutic benefit in this setting ^[102–104], [106,150–157]^.

### Pulmonary infections

Acute and chronic bacterial and viral pneumonias demand rapid cell autonomous and multicellular defense in the airways. Autophagy contributes to this defense by mediating selective antimicrobial clearance, but pathogens often subvert the pathway to their advantage^[14,163]^.

In *Mycobacterium tuberculosis* (*Mtb*) infection, interferon-gamma (IFN-γ) activates xenophagy through p62, NDP52, and OPTN, increasing phagolysosome fusion and killing^[164–166]^. However, virulent strains counteract this defense by activating AKT–mTOR signaling or disrupting HOPS–SNARE assembly, thereby stalling autophagosome maturation and converting autophagosomes into replication-permissive compartments^[167,168]^. Similarly, in alveolar macrophages infected with *Pseudomonas aeruginosa* or *Burkholderia cenocepacia*, failure of acidification and poor LC3 recruitment allow bacterial persistence and exacerbate inflammatory injury^[163,169,170]^.

Viral infections also exploit autophagy. Influenza is initially controlled by xenophagy and enhanced antigen presentation, but viral proteins can block fusion. In SARS-CoV-2 infection, ORF3a blocks HOPS-mediated SNARE assembly and other viral proteins impair lysosome acidification and reduce Beclin1, collectively stalling autophagy flux and facilitating viral replication^[171–173]^. These are direct mechanistic demonstrations that viruses can convert autophagy from defense into liability under defined conditions.

Autophagy also shapes innate effector responses: it primes neutrophils for extracellular trap formation, which can trap pathogens but also drive thrombosis and lung injury when unrestrained^[147,148]^. Modulating mitophagy and inflammasome components offers a route to rebalance pathogen control and tissue protection^[149,174]^.

From a therapeutic perspective, efficient xenophagy and phagolysosome acidification are essential for bacterial clearance. Thus, lysosomotropic agents such as HCQ and CQ, which raise lysosomal pH and block fusion, can antagonize host defense, consistent with mechanistic data and the mixed clinical outcomes of these drugs in viral pneumonia. In contrast, pharmacological approaches that restore or enhance flux without neutralizing lysosomes appear more aligned with antimicrobial objectives ^[102–104], [163–168]^.

### Lung cancer

Lung cancer is a leading cause of death arising in the pulmonary epithelium and evolving within the unique metabolic and immunologic microenvironments of the lung. It is also the lung disease with the most mature, lung-specific *in vivo* evidence that autophagy can be either protective or pathogenic depending on cell identity and disease stage. Including this section does not attempt a pan-cancer survey. Rather, it distills lung-specific mechanisms that exemplify the central theme of this review: cell compartment, micro-niche, and timing determine whether autophagy restores homeostasis or entrenches pathology^[175–177]^.

In premalignant epithelium, autophagy removes dysfunctional mitochondria and prevents p62 accumulation, limiting oxidative DNA damage and suppressing initiation^[178–181]^. Once KRAS-driven lung tumors are established, autophagy maintains tricarboxylic acid (TCA) cycle flux, nucleotide pools, and redox balance. In KP(KRAS;Trp53) and KL(KRAS;LKB1) lung cancer models, deleting Atg7 reduces tumor burden and produces oncocytoma-like lesions filled with defective mitochondria, demonstrating a direct dependence on autophagy for tumor maintenance *in vivo*^[181–188]^. LKB1 loss lowers metabolic plasticity and increases reliance on autophagy, creating a genetically defined vulnerability^[189,190]^. These lung-specific data anchor the broader message that autophagy is tumor suppressive in initiation yet tumor promoting in progression, and that the switch point is set by oncogene and tumor suppressor wiring and by metabolic demand.

Autophagy also shapes the pulmonary tumor microenvironment. In established non-small cell lung cancer (NSCLC), autophagy limits major histocompatibility complex (MHC) class I presentation and sustains resistance to epidermal growth factor receptor or anaplastic lymphoma kinase inhibitors. In LKB1 mutant tumors, inhibiting ULK1 restores antigen presentation and improves response to programmed cell death protein 1 (PD-1) blockade and to mitogenactivated protein kinase kinase (MEK) inhibitors, with *in vivo* evidence in lung models^[191–197]^. These findings illustrate why lung cancer is an instructive case for the whole review: they show, in the tissue of interest, that tumor intrinsic autophagy supports metabolism and immune escape, while host autophagy can either aid antitumor immunity or supply nutrients depending on context^[191,198]^. Clinically used HCQ has been combined with targeted and immune therapies to inhibit tumor autophagy, and reports of impaired response to anti-PD-1 in some models underscore the need for biomarker guided selection and for strategies that inhibit tumor autophagy while preserving host antitumor functions^[191,193,194,199]^.

### Cystic fibrosis (CF)

CF is a monogenic disorder of epithelial ion transport that produces dehydrated mucus, chronic infection, and progressive bronchiectasis with early life onset^[200]^. Cystic fibrosis transmembrane conductance regulator (CFTR) dysfunction increases ROS and activates transglutaminase 2 (TG2), which crosslinks Beclin1 and related proteins and sequesters them with p62 and histone deacetylases 6 (HDAC6) in aggresome-like structures. The Beclin1 and VPS34 complex is therefore inhibited, autophagosome formation and maturation are blocked, and proteotoxic stress persists with NF-κB activation^[201–204]^. These events have been demonstrated in patient-derived cells and experimental models.

CFTR deficiency also compromises xenophagy by reducing phagolysosome acidification and impairing LC3 recruitment to bacteria-containing vacuoles. This allows intracellular bacteria to persist while cGAS–STING and NLRP3 remain chronically activated. Epigenetic silencing of ATG12 further limits conjugation capacity, exacerbating defective autophagy^[169,170,205,206]^. IFN-γ priming or rapamycin treatment restores bacterial clearance, and CFTR modulators improve acidification and xenophagy^[169,206]^.

Therapeutically, cysteamine inhibits TG2 and releases Beclin1, while epigallocatechin gallate (EGCG) stabilizes rescued CFTR and improves lysosomal function. Together, these interventions convert a stalled program back into effective proteostasis and host defense, complementing the activity of CFTR correctors^[207–209]^. Mechanistically, cysteamine and EGCG rescue the Beclin1–VPS34 complex from transglutaminase-driven sequestration, restoring autophagy execution while improving CFTR trafficking antimicrobial defense ^[169,201–204], [207–209]^.

### Pulmonary hypertension (PH)

PH encompasses a spectrum of disorders marked by elevated pulmonary arterial pressure and progressive vascular remodeling that culminates in right ventricular failure^[210,211]^. In the pulmonary artery smooth muscle cells, hypoxia and metabolic stress activate AMPK/mTOR–regulated autophagy, which supplies metabolic substrates, preserves mitochondrial function, and supports the proliferation and migration that drive medial thickening. Pharmacologic or genetic inhibition of autophagy in this context reduces smooth muscle viability and increases apoptosis in monocrotaline-induced PH models^[212–215]^.

In contrast, basal endothelial autophagy preserves tetrahydrobiopterin synthesis and nitric oxide synthase coupling, restrains oxidative stress, and maintains barrier function^[216,217]^. Yet, under specific conditions and in defined vascular segments, chronic autophagy activation can promote maladaptive processes such as endothelial-to-mesenchymal transition and plexiform lesion formation. Transcriptional regulators including sirtuin 1 (SIRT1), forkhead box protein  O1 (FOXO1), hypoxia-inducible factor1α (HIF-1α), and TFEB establish these set points, while endothelial heterogeneity contributes to divergent outcomes^[218,219]^.

Defective autophagy that impairs lysosomal clearance augments NLRP3 activity and macrophage infiltration in remodeling vessels, and epigenetic modifiers such as HDAC, enhancer of zeste homolog 2 (EZH2), and microRNAs further tune ATG expression^[220–223]^. This complexity creates an asymmetric therapeutic logic: inhibiting smooth muscle autophagy may limit proliferation, whereas preserving basal endothelial autophagy is essential for vasodilation and redox balance ^[212–219, 223]^.

Currently, no autophagy-directed therapies are approved for PH. Given that smooth muscle cells rely on autophagy for stress survival, while endothelial cells require basal flux for nitric oxide coupling, nonspecific autophagy inhibition with lysosomotropic agents is unlikely to be beneficial. Instead, any strategy involving mTORC1 or AMPK modulation should be carefully designed to constrain smooth muscle autophagy while maintaining endothelial flux ^[106,212–219, 223]^.

## Therapeutic targeting of autophagy in lung disease

### Pharmacologic modulators of autophagy

Autophagy can be pharmacologically manipulated at multiple nodes of the pathway^[224]^. Canonical inducers include mTORC1 inhibitors such as rapamycin and metformin, which promote autophagosome initiation by relieving ULK1 inhibition^[106,107]^. Polyamines like spermidine induce autophagy via epigenetic regulation and have shown anti-inflammatory and anti-fibrotic effects in preclinical models^[225,226]^. Conversely, inhibitors such as HCQ, bafilomycin A1, and emerging VPS34 and ULK1 inhibitors block autophagosomelysosome fusion or autophagosome formation, thereby suppressing autophagic flux^[102–104]^.

These pharmacologic agents have been evaluated in disease-specific contexts: rapamycin attenuates fibrosis and inflammation in IPF models^[133]^; HCQ enhances tumor sensitivity to chemotherapy, targeted therapy and immunotherapy in KRAS-mutant lung cancer^[199]^; and cysteamine restores autophagy and improves CFTR trafficking in CF^[227]^. However, systemic modulation poses toxicity risks, emphasizing the need for context-specific, temporally controlled, and cell-targeted strategies^[228,229]^ (Table 1).

### Disease-specific therapeutic strategies and challenges

IPF: Autophagy is suppressed in IPF^[124]^. Reinstating flux via mTOR inhibition (rapamycin, metformin) or activating AMPK pathways attenuates fibroblast activation and extracellular matrix (ECM) deposition^[129]^. However, chronic or excessive autophagy activation may paradoxically support myofibroblast persistence or immune suppression^[230]^.

COPD: The dual role of autophagy in COPD requires nuanced modulation^[105]^. Early activation limits oxidative stress and senescence, whereas excessive or defective mitophagy exacerbates emphysema progression^[84,231]^. Selective restoration of basal autophagy in epithelial and immune cells may hold therapeutic value.

CF: Autophagy restoration through TGM2 inhibition (cysteamine), antioxidant therapy (EGCG), or nanoparticle-based delivery rescues CFTR trafficking and reduces inflammation^[207,209]^. Combining autophagy activation with CFTR correctors may yield synergistic benefits.

Lung Cancer: Autophagy supports established tumor survival, immune evasion, and resistance to therapy^[193,194]^. Inhibitors such as HCQ or ULK1/VPS34 inhibitors sensitize tumors to tyrosine kinase inhibitors (TKIs), Trametinib, and anti-PD-1 therapy^[175,196,232]^. Targeting tumor-intrinsic autophagy while preserving host defense is a key therapeutic challenge.

Infectious Lung Disease: Autophagy facilitates bacterial and viral clearance via xenophagy. However, some pathogens exploit autophagy to evade immunity^[17,233]^. Enhancing selective autophagy (e.g., via vitamin D3 or IFN-γ) may promote pathogen degradation without inciting immunopathology^[234]^.

PH: In PASMCs, excessive autophagy supports proliferation; whereas in endothelial cells, basal autophagy preserves vasodilatory function^[217,218]^. Cell-type-specific inhibition (e.g., with HDAC or miRNA-targeted strategies) may rebalance autophagy and restore vascular homeostasis^[235,236]^.

### Targeted delivery systems and spatial precision

Systemic modulation of autophagy carries the risk of off-target toxicity, underscoring the need for more precise and localized delivery strategies^[237]^. To address this, several therapeutic platforms are currently under investigation. Inhaled formulations of agents such as rapamycin, HCQ, and autophagy targeted nanoparticles allow direct delivery to the lungs, thereby reducing systemic exposure and minimizing adverse effects^[238–240]^. Nanocarriers designed for pH responsive or ligand guided delivery to specific intracellular compartments, such as lysosomes or mitochondria, enable precise targeting at the subcellular level^[241]^.

Genetic approaches using small interfering RNAs or CRISPR-Cas9-based systems to modulate key autophagy regulators such as Atg5, Beclin1, or PINK1 have shown promise in preclinical models^[242–244]^. However, their clinical application remains limited by challenges in delivery efficiency and potential immunogenicity^[245]^. Optimizing these delivery systems will be critical for translating autophagy targeted therapies into safe and effective treatments for lung diseases.

### Considerations for clinical translation

Despite encouraging results from preclinical studies, the clinical translation of autophagy modulators has been limited by several key challenges. One major barrier is the context-dependent nature of autophagy, which can exert either protective or pathogenic effects depending on the disease stage, cell type, and autophagic flux^[246,247]^. Additionally, there is a scarcity of reliable biomarkers for monitoring autophagy activity in human tissues. Commonly used indicators such as LC3-II turnover and p62 degradation are difficult to assess in vivo, particularly in clinical settings^[248,249]^.

Another concern is the therapeutic window. While tumors often exhibit heightened reliance on autophagy compared to normal tissues, offering a potential therapeutic advantage, prolonged systemic inhibition can result in detrimental effects such as cachexia, immunosuppression, and neurodegeneration^[250–252]^. Moreover, although autophagy inhibition has been shown to enhance the efficacy of tyrosine kinase inhibitors, mTOR inhibitors, and immunotherapies, the optimal dosing strategies and treatment sequences remain to be defined^[103,253]^.

To overcome these challenges, future progress will require the development of tools for real-time monitoring of autophagic flux, improved patient stratification based on disease-specific autophagy profiles, and precision targeting approaches guided by single cell and spatial multi-omics technologies.

## Emerging concepts and technologies

Technological progress is reshaping our view of autophagy as a spatially compartmentalized, tightly regulated, and selective degradative system. New methods now resolve autophagy dynamics in single cells and intact tissues and are revealing roles for selective autophagy in lung homeostasis and disease that were previously unappreciated.

### Single cell and spatial omics in autophagy research

Single-cell atlases show that autophagy programs vary by cell identity and state across epithelial, immune, endothelial, and mesenchymal lineages. Expression of core ATG modules, cargo receptors such as p62 and NBR1, and regulators such as TFEB and FOXO3 differs across lung compartments and disease phases, supporting a view of autophagy as a cellstate program rather than a single marker readout^[11,254,255]^.

Spatial transcriptomics and proteomics now map autophagyrelated pathways in situ. In PH, single cell and spatial profiling identify a microvascular endothelial subcluster at muscularized arterioles with a rewired autophagy signature and distinct sensitivity to perturbation, consistent with segmentspecific remodeling^[218,256]^.

In IPF, single-cell comparisons show variability in p62 and microtubule-associated protein 1 light chain 3 beta (MAP1LC3B) within transitional type two alveolar states and stromal subsets, while tissue studies demonstrate regions with high p62 and low LC3 puncta that indicate stalls in flux. These observations motivate integrated spatial proteotranscriptomic approaches that combine LC3 and p62 immunostaining with spatial RNA to resolve throughput versus expression^[236,257]^.

In COPD, high-resolution atlases of the small airway identify epithelial and immune microniches that drive emphysema. Their open matrices can be scored for ATG core, BNIP3 and BNIP3L-indexed mitophagy, and TFEB lysosome target sets across basal, ciliated, secretory, and alveolar attachment cells to generate hypotheses about ciliophagy and mitophagy failure zones^[258–261]^.

Paired single cell and spatial atlases in infection and acute injury map regional epithelial injury, macrophage-epithelium crosstalk, and expansion of fibrogenic stroma. These datasets can be mined for xenophagy and mitophagy modules in infected epithelium versus bystander cells^[34,262,263]^.

Resected NSCLC reveal spatially distinct tumor, stroma, and immune neighborhoods. Autophagy-linked gene modules that include ULK1 and ATG13, the VPS34 axis, TFEB programs, and ferritinophagy or mitophagy signatures localize tumor-intrinsic autophagy dependence at invasive fronts and outline stromal nutrient circuits^[264–266]^.

A consistent lesson across lung datasets is anatomical polarity. Cell identity, microniche, and disease timing together determine whether autophagy restores homeostasis or entrenches pathology. These insights support the development of spatially anchored, flux-aware biomarkers to guide therapy^[34,254–266]^.

### In vivo imaging of autophagy flux

Direct imaging of autophagy throughput in living tissues is critical for translational progress. Dual-labeled LC3 reporters, such as mCherry-GFP-LC3, enable distinction between autophagosomes and autolysosomes based on pH-sensitive fluorescence^[258,259]^. When expressed in transgenic mice or delivered by viral vectors, these reporters enable visualization of autophagy flux within defined lung compartments. Intravital microscopy combined with biosensors or metabolic tracers such as uniformly labeled glucose allows monitoring of autophagy-mediated substrate recycling in tumor or fibrotic regions of the lung^[261,263]^. These approaches reveal how spatial and temporal heterogeneity in autophagy flux correlates with outcomes including survival, inflammatory signaling, and metabolic rewiring.

### Expanding the scope of selective autophagy

Autophagy is not only a bulk degradative process. It also comprises selective recycling programs that are tuned to organelles, macromolecules, or pathogens. These specialized forms are increasingly implicated in lung diseases. Mitophagy, which eliminates damaged mitochondria, is altered in COPD, asthma, and lung cancer^[61,262]^. Lipophagy governs turnover of lipid droplets and fatty acid oxidation and is impaired in PH and in tumors driven by KRAS^[34,265]^. Ferritinophagy supports iron sulfur cluster biogenesis in mitochondria and contributes to metabolic adaptation in cancer cells^[266]^. ER-phagy clears damaged or excess ER during chronic stress or viral infection^[116]^. Xenophagy eliminates intracellular pathogens yet is often hijacked by organisms that include *Mtb* and severe acute respiratory syndrome coronavirus^[2,267]^.

Each selective pathway is mediated by distinct cargo receptors that confer specificity. For example, NCOA4 directs ferritinophagy^[268]^, BNIP3L regulates mitophagy^[269]^, and FAM134B mediates ER-phagy^[270]^. Importantly, these pathways are regulated in a disease-specific manner. Therapeutic interventions that target these selective routes may therefore achieve greater precision and efficacy while preserving the basal autophagy that is essential for cellular homeostasis.

### Nextgeneration, selective autophagy targeting

New chemical platforms now enable selective degradation of disease-associated proteins or organelles through autophagy. Autophagy-tethering compounds (ATTECs) are small molecules that simultaneously bind LC3 and a designated target, thereby directing that target into autophagosomes for degradation^[271]^. Autophagy-Targeting Chimeras (AUTACs) are chimeric molecules that install K63 linked ubiquitin to recruit p62 and promote delivery to autophagosomes^[272]^. Autophagy-Targeting Chimeras via p62 (AUTOTACs) engage the ZZ domain of p62 without requiring ubiquitination and thus broaden the spectrum of druggable substrates^[273]^. These approaches achieve target-specific degradation without inducing cellular stress and hold the promise of reducing off-target effects. Layering these chemistries onto spatial omics may help identify patient-specific degradation signatures and advance personalized therapeutic strategies.

This logic can be applied to pulmonary disease contexts. In lung cancer, tumor cells often exhibit autophagy addiction, which is further accentuated by loss of LKB1. Candidate tumor-intrinsic targets include ULK1 or ATG7, to collapse initiation; glucose-6-phosphate dehydrogenase (G6PD) to deplete nicotinamide adenine dinucleotide phosphate (NADPH) and redox buffering capacity; and stearoylCoA desaturase1 (SCD1) or acetylCoA acetyltransferase1 (ACAT1) to disrupt lipid-supported oxidative metabolism^[196,274,275]^. Spatial profiling can pinpoint high ULK1 and TFEB activities at invasive fronts and nominate patients for regimens that focus on ULK1 with AUTOTACs. Potential pharmacodynamic readouts include the collapse of the TCA flux, accumulation of lipid droplets in oncocytoma-like regions, and restoration of MHC class I-mediated antigen presentation^[276,277]^.

In fibrotic epithelium and stroma, suppressed autophagy coexists with persistent TGF-β and mTORC1 signaling^[230]^. Druggable reinforcement nodes include Yes-associated protein (YAP)/transcriptional coactivator with PDZ-binding motif (TAZ) and the NADPH oxidase4 (NOX4), which sustain myofibroblast programs^[278,279]^. ATTECs that degrade YAP, or AUTACs that tag NOX4, could blunt profibrotic transcription and redox drivers in fibroblasts. In parallel, tethers bias toward ER-phagy may contract the expanded secretory ER in AT2 cells, thereby reducing export of profibrotic cytokines^[280,281]^. Tissue biomarkers that signal target engagement include p62 accumulation with low LC3 puncta in fibrotic foci, high mTORC1 activity whithin fibroblast clusters, and a decline in collagen translation^[282]^.

Cigarette smoke induces mitochondrial injury and disrupts autophagic flux in airway epithelial cells and alveolar macrophages. Organelle-directed ATTECs that selectively remove damaged mitochondria may lower mitochondrial ROS and NLRP3 activation. Similarly, tethers that remove lipid droplets may reverse lipotoxicity and mucostasis^[283]^. In parallel, AUTACs designed to tag NLRP3 or gasdermin D (GSDMD) for p62-dependent delivery offer a way to limit pyroptosis and release of damage-associated molecular patterns (DAMPs) without broadly suppressing innate defense. Pharmacodynamic assessment of on-target activity may include reduced cytosolic mtDNA, decreased IL-1β release, and restoration of ciliary beat indices^[284,285]^.

Hyperinflammation and barrier failure are amplified by NLRP3 inflammasomes and GSDMD pores^[286]^. Emerging chemical platforms align with the known role of autophagy in restraining pyroptosis in alveolar macrophages and protecting epithelial and endothelial barriers. For example, AUTACs that install K63 linked ubiquitin on NLRP3 or ASC, and AUTOTACs that direct GSDMD to autolysosomes, could selectively suppress pyroptotic signaling^[287]^. A flux-aware regimen should be timed to the hyperinflammatory phase and monitored by declines in caspase-1 activity and IL-1β release together with improvement in epithelial tight junction integrity^[288]^.

In CF, a feedback loop between ROS and TG2 sequesters Beclin1 and stalls execution of autophagy^[170]^. AUTOTACs directed to TG2 could relieve the block and restore the Beclin1 and VPS34 complex, which would complement modulators of CF transmembrane conductance regulator^[289]^. Alternative epithelial targets include HDAC6, which dismantles aggresomes, and peroxisome proliferator activated receptor gamma (PPARγ) aggregates, which sustain NF-κB activation^[290]^. Biomarkers of on-target activity include the liberation of Beclin1 from aggresomes, the restoration of LC3 flux, and the improved surface expression of CFTR^[169,202,203,207,208]^.

In PH, hypoxia-driven autophagy in PASMCs sustains proliferation and migration. Candidate cargo for selective degradation includes HIF-1α, forkhead box M1 (FOXM1), and platelet-derived growth factor receptor β (PDGFRβ)^[291,292]^. Removing these proteins via AUTACs delivered by inhaled, vessel-targeted nanoparticles could reduce medial thickening while preserving the basal endothelial autophagy needed for nitric oxide coupling^[219]^. Response metrics include lower Ki-67 expression in smooth muscle clusters, restoration of nitric oxide bioavailability, and decreased muscularization in distal vessels^[293]^.

In type 2-dominant asthma, excessive epithelial autophagy supports mucin biogenesis and barrier dysfunction^[294]^. AUTACs targeting SAM pointed domain-containing ETS transcription factor (SPDEF) or the ER chaperone anterior gradient2 (AGR2) could reduce MUC5AC production while preserving host defense^[295]^. In steroid-resistant phenotypes with high inflammasome activity, selective removal of NLRP3 in macrophages with AUTACs may restore steroid sensitivity and lower exacerbation risk^[296]^. Efficacy could be tracked by mucus plug indices, transepithelial electrical resistance of the epithelium, and sputum IL-1β^[297]^.

In pulmonary infections, selective autophagy modulation also holds promise. During bacterial pneumonia, AUTACs that tag ubiquitinated phagosomal cargo or Rab7 cofactors could enhance xenophagy in alveolar macrophages against *Mtb* and opportunists such as *Pseudomonas*^[298]^. During viral pneumonia, AUTACs directed against SARS-CoV-2 proteins such as ORF3a, which block autophagosome-lysosome fusion and lysosomal acidification, could neutralize autophagy antagonists and restore flux in infected epithelium, with careful staging to avoid suppression of antiviral immunity^[173]^. Readouts include increased LC3-LAMP1 colocalization, reduced viral load, and lower biomarkers of neutrophil extracellular traps^[299]^.

Across these contexts, a practical framework for target amenability emerges. Candidate substrates should (i) present a cytosolic face that allows LC3 tethering or p62 engagement, (II) occupy a causal position in the relevant lung cell type and disease stage so that degradation produces correction rather than compensation, and (iii) show spatially restricted expression that can be exploited by inhaled delivery or ligand based targeting to minimize off-tissue effects. Early-phase clinical testing should integrate pharmacodynamic readouts, such as combined p62 and LC3 staining with TFEB localization, together with disease-specific functional outcomes^[300,301]^.

### Computational modeling and systems integration

Existing single-cell and spatial matrices from COPD, PH, IPF, and NSCLC already enable scoring of autophagy-related modules. These include core ATG machinery, mitophagy programs such as PINK1-PRKN or BNIP3-BNIP3L, and lysosome or TFEB, TFE3 transcriptional signatures^[255,302]^. Such scores can be mapped onto anatomical neighborhoods and cell-to-cell circuits to reveal microenvironments that favor or suppress autophagy. In lung cancer, a combined cell death index that includes autophagy and necroptosis features has been built from bulk, single-cell, and spatial transcriptomes, and shown to stratify prognosis^[303]^. Similarly, neighborhood graphs and ligand-receptor maps from COVID-19 lung atlases and pulmonary hypertension endothelial datasets overlay autophagy modules with cytokine milieus, for example, IFN-γ and IL-1β rich regions that coincide with p62 accumulation^[304]^. While these applications demonstrate feasibility and biological association, they have not yet been standardized for prospective patient selection^[305,306]^.

The next steps for flux-aware computational modeling are practical. First, co-register spatial RNA with spatial proteomics to quantify LC3 puncta, p62 aggregates, LAMP1/2-defined lysosomal area, and nuclear localization of TFEB or TFE3. These readouts can be integrated into a FluxScore for each microniche^[307]^. Second, use mechanism-anchored digital pathology to train models on paired hematoxylin-eosin and immunofluorescence images. By quantifying the autophagosome density, p62 aggregates, and lysosomal competence, image-derived scores can be linked single-cell neighborhoods to predict therapeutic sensitivity, for example, to ULK1 inhibition in LKB1-deficient lung cancer^[308]^. Third, combine Perturb-seq with spatial readouts in organoids or in precision-cut lung slices, editing key regulators such as ULK1, ATG7, TFEB, or BNIP3L. These perturbations can establish causal circuits, such as endothelial autophagy that drives smooth muscle muscularization, or AT2 cell autophagy that primes fibroblast activation^[218]^.

Collectively, these steps require harmonized spatial proteomics, preregistered analysis pipelines, and prospective validation that is embedded in biomarker-driven clinical studies. Together, they define a realistic trajectory from descriptive association to predictive modeling of autophagy in pulmonary disease^[305,306]^.

## Conclusions and future perspectives

Autophagy is a fundamental homeostatic mechanism that governs pulmonary cell survival, metabolic fitness, immune regulation, and organ integrity. Its role in the lung is highly context- and cell-type dependent: protective in normal homeostasis and acute stress, but potentially pathogenic or tumor-promoting when dysregulated or co-opted by disease processes.

Across a range of lung diseases, including fibrosis, cancer, infection, COPD, CF, and PH, autophagy plays diverse and context-dependent roles. In certain conditions, autophagy suppression promotes disease progression by inducing mitochondrial dysfunction, protein aggregation, or impaired pathogen clearance. In contrast, sustained activation or impaired completion of autophagy can contribute to pathological tissue remodeling, immune evasion, and resistance to therapy.

Importantly, a disease-specific vulnerability to autophagy modulation has been identified. KRAS driven lung tumors with loss of LKB1 or TP53 exhibit metabolic dependence on autophagy, whereas fibrotic lungs show reduced autophagic flux that increases their sensitivity to mTOR inhibition or AMPK activation. In addition, autophagy deficiency in the host, independent of tumor cells, disrupts systemic nutrient availability such as arginine and alanine, and enhances interferon-mediated antitumor immune responses. These findings highlight the broader systemic role of autophagy in lung disease pathogenesis.

The therapeutic question is therefore not *whether* autophagy should be targeted, but *how*. Global inhibition poses risks, such as neurodegeneration, cachexia, and immunosuppression. In contrast, context-specific modulation, including the enhancement of autophagy in degenerative or infectious diseases while constraining it in cancer or fibrosis, requires precise spatial and temporal control.

To achieve these goals, future research should prioritize several areas. First, dissect compartmentalized autophagy functions across lung cell types using single-cell and spatial multi-omics. Second, develop biomarkers and surrogate readouts that faithfully reflect autophagic flux in human tissues. Third, design targeted delivery systems to restrict autophagy modulation to diseased lung regions and minimize off-target effects. Fourth, integrate autophagy profiling into patient stratification and therapeutic decision-making to advance precision medicine.

In addition, age is a major determinant of autophagy capacity. The natural decline in autophagy in aging lungs may increase vulnerability to fibrosis, infection, and injury^[309]^. Therapeutic strategies aimed at restoring autophagy in elderly individuals, particularly when combined with senolytic, metabolic, or immunomodulatory interventions, have the potential to improve pulmonary resilience and tissue repair^[8,310,311]^.

In conclusion, autophagy is not a uniform or universally applicable therapeutic target. It is a dynamic, adaptable regulator of lung health and disease whose safe and effective therapeutic exploitation will require precision, personalization, and deep mechanistic insight.

## Abbreviations used in this review

ALI: acute lung injury
ALK: anaplastic lymphoma kinase
AMBRA1: autophagy and Beclin–1 regulator–1
AMPK: AMP–activated protein kinase
ASC: apoptosis–associated speck–like protein containing a CARD
AT2: alveolar type II (cell)
ATG: autophagy–related gene (family)
ATTECs: autophagy–tethering compounds
AUTACs: autophagy–targeting chimeras
AUTOTACs: autophagy–targeting chimeras via p62
BECN1: Beclin–1
BNIP3: BCL2/adenovirus E1B 19 kDa–interacting protein–3
BNIP3L (NIX): BNIP3–like protein (also NIX)
CARD: caspase activation and recruitment domain
CF: cystic fibrosis
CFTR: cystic fibrosis transmembrane conductance regulator
COPD: chronic obstructive pulmonary disease
cGAS: cyclic GMP–AMP synthase
DAMPs: damage–associated molecular patterns
DFCP1: double FYVE domain–containing protein–1
EGCG: epigallocatechin gallate
EGFR: epidermal growth factor receptor
EMT: epithelial–mesenchymal transition
ENOS (eNOS): endothelial nitric oxide synthase
ER: endoplasmic reticulum
EZH2: enhancer of zeste homolog–2
FAM134B: family with sequence similarity–134 member–B (ER–phagy receptor)
FOXM1: forkhead box M1
FOXO1: forkhead box O1
G6PD: glucose–6–phosphate dehydrogenase
GABARAP: γ–aminobutyric acid receptor–associated protein
GSDMD: gasdermin–D
HCQ: hydroxychloroquine
CQ: chloroquine
HDAC/HDAC6: histone deacetylase / histone deacetylase–6
HIF–1α: hypoxia–inducible factor–1α
HOPS: homotypic fusion and vacuole protein sorting (tethering complex)
IFN–γ: interferon–γ
IL–#: interleukin–#, e.g., IL–1β, IL–4, IL–10, IL–13, IL–18, IL–25, IL–33
IPF: idiopathic pulmonary fibrosis
KL: KRAS;LKB1 genotype
KP: KRAS;TP53 genotype
LAMP1/2: lysosome–associated membrane protein–1/2
LC3: microtubule–associated protein–1 light chain–3
LKB1: liver kinase B1 (STK11)
LPS: lipopolysaccharide
MEK: mitogen–activated protein kinase kinase
MHC: major histocompatibility complex
MLKL: mixed lineage kinase domain–like pseudokinase
Mtb: Mycobacterium tuberculosis
mTOR: mechanistic target of rapamycin
mTORC1: mechanistic target of rapamycin complex–1
MyD88: myeloid differentiation primary response–88
NADPH: nicotinamide adenine dinucleotide phosphate (reduced)
NBR1: neighbor of BRCA1 gene–1
NDP52 (CALCOCO2): nuclear dot protein–52
NF–κB: nuclear factor kappa–light–chain–enhancer of activated B cells
NLRP3: NOD–like receptor family pyrin domain–containing–3
NOX4: NADPH oxidase–4
NSCLC: non–small cell lung cancer
OPTN: optineurin
ORF3a: open reading frame–3a (of SARS–CoV–2)
ORMDL3: ORMDL sphingolipid biosynthesis regulator–3
PASMC(s): pulmonary artery smooth muscle cell(s)
PD–1: programmed cell death protein–1
PDGFRβ: platelet–derived growth factor receptor–β
PI3K: phosphatidylinositol 3–kinase
PIK3C3/VPS34: class III PI3K catalytic subunit type–3 / vacuolar protein sorting–34
PINK1: PTEN–induced kinase–1
PRKN: parkin RBR E3 ubiquitin protein ligase (parkin)
RAB7: Ras–related protein Rab–7 (small GTPase)
RIPK1/3: receptor–interacting serine/threonine–protein kinase–1/–3
ROS: reactive oxygen species
SARS–CoV–2: severe acute respiratory syndrome coronavirus–2
SCD1: stearoyl–CoA desaturase–1
SIRT1: sirtuin–1
SNARE: soluble N–ethylmaleimide–sensitive–factor attachment protein receptor
SPDEF: SAM pointed domain–containing ETS transcription factor
SQSTM1/p62: sequestosome–1 (p62)
STING: stimulator of interferon genes
STX17: syntaxin–17
TAZ: transcriptional co–activator with PDZ–binding motif
TCA: tricarboxylic acid (cycle)
TFEB/TFE3: transcription factor EB/E3
TGF–β: transforming growth factor–β
TKIs: tyrosine kinase inhibitors
TLR4: Toll–like receptor–4
TNF–α: tumor necrosis factor–α
ULK1: Unc–51–like autophagy activating kinase–1
USP13: ubiquitin–specific peptidase–13
VAMP8: vesicle–associated membrane protein–8
V–ATPase: vacuolar H^+^–ATPase
WIPI2: WD–repeat phosphoinositide–interacting protein–2
YAP: Yes–associated protein

## Data Availability

Data sharing is not applicable to this article as no data were created or analyzed in this study.
